# Integrating regional and community lung cancer services to improve patient care

**DOI:** 10.3747/co.2007.157

**Published:** 2007-12

**Authors:** M. Dahele, Y. Ung, J. Meharchand, H. Shulman, R. Zeldin, A. Behzadi, C. Simone, S. Cheng, C. Weigensberg, K. Sivjee

**Affiliations:** * Department of Radiation Oncology, Odette Cancer Centre and University of Toronto, Sunnybrook Health Sciences Centre, Toronto, Ontario; † Department of Medical Oncology, Toronto East General Hospital, Toronto, Ontario.; ‡ Department of Medical Imaging, Sunnybrook Health Sciences Centre and University of Toronto, Toronto, Ontario.; § Department of Thoracic Surgery, Toronto East General Hospital and University of Toronto, Toronto, Ontario.; || Department of Thoracic Surgery, The Scarborough Hospital and University of Toronto, Scarborough, Ontario.; # Department of Medical Oncology, Odette Cancer Centre and University of Toronto, Sunny-brook Health Sciences Centre, Toronto, Ontario; ** Department of Pathology, The Scarborough Hospital, Scarborough, Ontario; †† Department of Respirology, Sunnybrook Health Science Centre and University of Toronto, 2075 Bayview Avenue, Toronto, Ontario, Canada M4N 3M5

**Keywords:** Health care organization, lung cancer, multidisciplinary team, multidisciplinary clinic, clinical network, tumour board

## Abstract

Lung cancer is the leading cause of cancer death in Canada. The organization of health care services is central to the delivery of accessible, high-quality medical care and may be one factor that influences patient outcome. An exciting opportunity arose for clinicians to initiate the redesign of lung cancer services provided by three institutions in the Greater Toronto Area. This qualitative report describes the integrated lung cancer network that they developed, the innovation it has facilitated, and the systematic approach being taken to evaluate its impact. Available clinical resources were deployed to restructure services along patient-centred lines and to provide greater access to the specialist lung cancer team. A non-hierarchical clinical network was established that consolidates the lung cancer team. A multi-institutional and multidisciplinary tumour board and comprehensive thoracic oncology clinics are at its core. This innovative organizational paradigm considers all of the available services at each facility and aims to fully integrate specialists across the three institutions, thereby maximizing resource utilization. We believe that this paradigm may have wider applicability. The network is currently working to complete a current program of further service improvements and to objectively assess its impact on patient outcome.

## 1. INTRODUCTION

### 1.1 Demographics and Treatment

Lung cancer is the leading cause of cancer death in Canada, and overall survival has changed little in recent decades. In 2007, an estimated 23,300 new patients will be diagnosed, and 19,900 lung cancer patients will die. For patients diagnosed in 1996–1998, the age-standardized 5-year relative survival ratio in Ontario was 16%. About 80% of patients develop non-small-cell lung cancer (nsclc). Patients with operable early-stage nsclc have the best prognosis, but most patients present with locally advanced or metastatic nsclc, whose 5-year survival rates are approximately 10% and 5% respectively. Interest is growing among specialists, patients, and providers concerning the organization and design of lung cancer services, and the effect that improvements might have on patient outcomes, including survival^1^.

### 1.2 Lung Cancer Services

Despite conflicting evidence, data are available to suggest links between tumour size, treatment intent, and prognosis^2^. Prolonged patient journeys may therefore carry a risk of lung cancer progression that could affect treatment options^3^. Acknowledging that appreciable waits have been documented for Canadian lung cancer patients requiring surgery or radiotherapy, the *Ontario Cancer Plan 2005–2008* 4 includes development of increased capacity and of rapid-access strategies. Substantial investment in capital projects has occurred, including construction of several community cancer centres with radiotherapy facilities that will bring treatment closer to home for many patients. Improvements have already been seen in wait times for radiation treatment and cancer surgery.

The present report describes an innovative reorganization undertaken by colleagues at the Odette Cancer Centre (occ—formerly the Toronto–Sunnybrook Regional Cancer Centre) and two university-affiliated community teaching hospitals located in Ontario, the Toronto East General Hospital (tegh—approximately 9 km southeast of occ) and The Scarborough Hospital (tsh—approximately 14 km east of occ), to improve delivery and accessibility of specialist lung cancer services.

## 2. REDESIGNING LOCAL LUNG CANCER SERVICES

### 2.1 The Impetus

The occ is part of Sunnybrook Health Sciences Centre (shsc). The sixth-largest comprehensive cancer centre in North America, occ is one of two regional cancer centres in Toronto. It provides expertise in medical and radiation oncology and access to diagnostic and medical specialists. Lung cancer surgery for patients in the occ catchment area is performed at several hospitals. In addition, cross-appointed thoracic surgeons from tegh can also see and operate on patients at shsc. Most patients fall within the catchment areas of the tegh and tsh, both of which have thoracic surgeons, medical oncologists, and respirology and diagnostic services on site and both of which refer their patients to occ for radiotherapy.

Recently, clinical colleagues from the foregoing institutions agreed that the lung cancer services they provide would benefit from consolidation and that the process would afford an opportunity to strengthen patient-centred care. The interdependence and reliance of these three institutions indicated excellent suitability for a collaboration. The integrated service aimed to

- be patient-centred, to have an academic focus, and to make use of available resources to facilitate decision-making and to provide access to evidence-based multidisciplinary lung cancer care.
- provide a coordinated patient journey from initial suspicion to diagnosis and treatment of lung cancer.
- increase the number of patients seen when their disease is in its early stages and when they are candidates for curative-intent treatment, and to increase the opportunities to offer patients clinical trials and studies.

### 2.2 The Model

**FIGURE 1:**
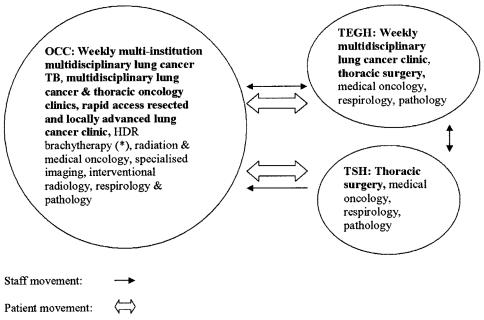
Multi-institutional lung cancer service model. Links with primary care are being established to fast-track the referral of patients with suspected lung cancer. The model also facilitates *ad hoc* surgical cooperation between Toronto East General Hospital (tegh) and The Scarborough Hospital (tsh). occ = Odette Cancer Centre (formerly the Toronto–Sunnybrook Regional Cancer Centre); tb = tumour board; hdr = high dose rate.

Proponents of the consolidation felt that a multi-institutional, multidisciplinary lung cancer tumour board (lctb), multidisciplinary lung cancer clinics, and strengthened links between the specialist team and primary care were all central to achieving the stated aims. Delineating a clear organizational structure, placing the multidisciplinary lctb at the centre of the service, and formally linking the thoracic surgery centres with the regional cancer centre (Figure 1) all meet specific recommendations made in both the Thoracic Surgical Oncology and Multidisciplinary Cancer Conference Standards which are produced by Cancer Care Ontario’s Program in Evidence-Based Care 5.

The weekly lctb started at occ in May 2005 and is held at a time that allows for regular attendance by specialists from occ, tegh, and tsh. It is accredited for continuing medical education through the Royal College of Physicians and Surgeons of Canada. Most of the cases discussed involve patients who might be candidates for radical multimodality treatment or whose disease poses clinical challenges. When appropriate, patients are considered for clinical trials. For example, in the 11 months before and after the lctb started meeting, 14 and 32 patients respectively were enrolled in the group’s highest-recruiting nsclc study. An average of about 10 cases are discussed each week, and all institutions are well represented (about 55% of cases from occ, 25% from tegh, and 20% from tsh).

The second strand of the remodelled service is weekly combined lung cancer clinics. In these clinics, patients can be simultaneously evaluated by multiple specialities, providing optimal management. To improve patient access, the clinics take place at tegh and occ. All occ staff can now refer patients with malignant thoracic disease (including metastases) to the tegh/shsc surgeons who staff the clinic. A number of thoracic surgeons also take part in the high dose rate brachytherapy service at occ.

## 3. FURTHER IMPROVEMENTS

Several additional improvement projects are currently underway:

- A time-to-treatment initiative with a consolidated referral pathway is being implemented between family medicine, respirology (occ), and thoracic surgery (tegh) for patients with suspected lung cancer. A patient-flow coordinator provides the next available consultation with an appropriate specialist closest to the patient and can book investigations using dedicated imaging slots. The impetus for this consolidation was the success of earlier redesign work carried out at tegh that had reduced to 30 from 128 days the overall time from suspicious chest radiograph to diagnosis 6. A Web-based database will record patient care timelines, clinical management details, and outcomes from this initiative. The rollout will include interactive education meetings with primary care physicians.
- A weekly rapid-access multidisciplinary clinic specifically for patients with resected and locally advanced lung cancer has recently been established to fast-track decision-making and to coordinate multimodal treatment in patients who may be being managed with curative intent. This clinic model is new. Wait times are 7 days or fewer, with urgent cases often being seen within 48 hours. Patient and staff satisfaction will be assessed, and information resources specific to these patient groups are currently being developed.
- A lctb database has been developed that will provide searchable information, including a record of participants, patient demographics, team decisions, and changes to provisional management plans, diagnosis, and staging. Thus, objective data is being gathered that will permit assessment of the effect of the lctb.
- A study is underway to compare cohorts of patients referred to lung cancer team members before and after initiation of the service changes. Journey times and outcomes are among the variables that are being studied. This ongoing work will generate quantitative data that will help to benchmark the service, to assess the effect of service changes on patient outcome, and to provide an impetus for continued improvement. That study will be the subject of a future publication.

## 4. CONCLUSION

Lung cancer management is challenging. Current medical systems have long been criticized for being fragmented and must evolve to deliver contemporary lung cancer treatment to as many patients as possible. The process of integrating clinical services across three independent and geographically separate facilities into a clearly defined service model has so far succeeded in part because it has been clinician-led with institutional support and buy-in, because it is inclusive, and because it has resulted in improvements for patients and physicians. A number of features distinguish this network:

- a non-hierarchical structure that breaches barriers and encourages cooperation,
- a two-way flow of personnel, and
- a multi-institutional lctb.

We believe that this paradigm may find wider application in the organization of cancer services.

The service model seeks to establish links with primary care, to manage patient referrals to the hospital-based lung cancer team, to provide local access to multidisciplinary lung cancer clinics, to offer a multidisciplinary rapid-access clinic for potentially curable patients, to aid knowledge transfer, to facilitate working to generally agreed guidelines, and to benefit clinical trial recruitment. It is also focused on gathering relevant outcome data to support service improvement. To date, the reorganization has required considerable time and effort, and many ongoing challenges remain, including the delivery of local multidisciplinary services to an ethnically and economically diverse population. The success of the integrated lung cancer service rests firmly on the hard work and commitment of all its staff.

Health care systems require continuous effort if delivery of care is to be enhanced and sustained in an increasingly challenging environment. However, gains can potentially be made by incorporating ideas from other sectors 7 and mechanisms to rapidly share and adopt ideas and successes 8. Specific examples of improvement methodologies, success stories, and access to improvement communities can be found on both the U.K. National Health Service Institute for Innovation and Improvement and the North American Institute for Healthcare Improvement Web sites.

## References

[1] Spear SJ (2005). Fixing health care from the inside, today. Harv Bus Rev.

[2] Port JL, Kent MS, Korst RJ, Libby D, Pasmantier M, Altorki NK (2003). Tumor size predicts survival within stage ia non-small cell lung cancer. Chest.

[3] O’Rourke N, Edwards R (2000). Lung cancer treatment waiting times and tumour growth. Clin Oncol (R Coll Radiol).

[4] Cancer Care Ontario (2004). Ontario Cancer Plan 2005–2008.

[5] Cancer Care Ontario (2007). Home | Standards & guidelines | Practice guidelines (pebc) [Web page].

[6] Lo DS, Zeldin RA, Skrastins R (2007). Time to treat: a system redesign focusing on decreasing the time from suspicion of lung cancer to diagnosis in a community hospital. J Thorac Oncol.

[7] Toyota Motor Corporation Home page | Company | Vision & philosophy | Toyota Production System [Web page]. www.toyota.co.jp/en/vision/production_system/.

[8] National Health Service (nhs) Institute for Innovation and Improvement (2007). Home page [Web page]. www.institute.nhs.uk/.

